# The role of soil microbiota in the control of parasitic weeds

**DOI:** 10.1093/pcp/pcaf125

**Published:** 2025-10-04

**Authors:** Pornkanok Pongpamorn, Michelle Zwart, Harro J Bouwmeester

**Affiliations:** Plant Hormone Biology Group, Green Life Sciences Cluster, Swammerdam Institute for Life Science, University of Amsterdam, Science Park 904, Amsterdam 1098 XH, The Netherlands; Plant Hormone Biology Group, Green Life Sciences Cluster, Swammerdam Institute for Life Science, University of Amsterdam, Science Park 904, Amsterdam 1098 XH, The Netherlands; Plant Hormone Biology Group, Green Life Sciences Cluster, Swammerdam Institute for Life Science, University of Amsterdam, Science Park 904, Amsterdam 1098 XH, The Netherlands

**Keywords:** biocontrol, Orobanchaceae, parasitic plants, plant growth-promoting fungi, plant growth-promoting rhizobacteria, plant–microbe interactions

## Abstract

Parasitic weeds from the Orobanchaceae family, particularly *Striga*, *Orobanche*, and *Phelipanche* spp., are responsible for substantial agricultural losses worldwide. A better understanding of the intricate chemical interaction between parasitic plants and their host crops, and the effect the rhizosphere microbiome may have on this, offers potential for developing sustainable and effective biocontrol strategies. We review the biology of parasitic plants, with a focus on host-derived signaling molecules such as strigolactones and haustorium-inducing factors that coordinate key stages of their lifecycle, and hence are potential targets for control through microorganisms. We highlight several examples of pathogenic microorganisms and plant growth-promoting rhizobacteria and fungi that have been shown to suppress parasitic weeds. These microbes act through multiple mechanisms: direct antagonism of the parasite, enhancement of the host’s defense responses, and interference with chemical signaling between host and parasite. Both laboratory and field studies are reviewed to evaluate the efficacy and future potential of these biological control agents.

## Introduction

Parasitic plants of the *Orobanchaceae* family, primarily broomrapes (*Orobanche* and *Phelipanche* spp.) and witchweeds (*Striga* spp.), cause severe agricultural losses globally. *Orobanche* and *Phelipanche* spp. are obligate root holoparasites that affect a wide range of crops including legumes (e.g. chickpea, pea, lentil, broad bean, and common vetch), vegetable crops (e.g. tomato, cabbage, carrot, eggplant, parsley, and watermelon), and oil crops (e.g. sunflower and oilseed rape) across Europe, the Middle East, and parts of Asia (Mutuku et al. 2021). In contrast, *Striga* spp. are obligate root hemiparasites that threaten cereal crops, such as sorghum, maize, pearl millet, and rice, as well as the legume cowpea in sub-Saharan Africa (Erdoğan 2021). The economic impact is considerable, with annual losses due to *Striga* infestations reaching an estimated US$7 billion in sub-Saharan Africa, specifically US$1.2 billion in Nigeria, US$87 million in Mali, and US$75 million in Ethiopia (Sibhatu 2016). *Striga* is most prevalent in Africa because many African soils are severely deprived of essential nutrients, which leads to increased exudation of host signaling molecules that stimulate *Striga* seed germination (Yoneyama et al. 2007, Ayongwa 2011, Jamil et al. 2011, 2013, Mandumbu et al. 2019). Moreover, the warm, semi-arid climate and erratic rainfall patterns across much of sub-Saharan Africa create favorable conditions for *Striga* development and allow the hundreds of thousands of seeds produced by a single plant to remain viable in the soil for over two decades (Mandumbu et al. 2019).

Conventional control strategies for parasitic plants include cultural, chemical, and genetic approaches (Sibhatu 2016). Trap crops are a cultural method that uses non-host plants to induce seed germination without allowing the parasite to develop, thus provoking suicidal germination. Although this is an effective way to deplete the parasitic seed bank, this method often requires skipping the main crop and investing in maintaining the trap crop, which can lead to disappointing returns (Jamil et al. 2021, David et al. 2022). Hand weeding is another cultural method, but it is labor-intensive and usually too late to prevent crop damage since parasitic plants harm the host already before they emerge. Applying fertilizers with phosphorus and nitrogen can also suppress *Striga* and promote crop growth, but this option is expensive and out of reach for most African smallholder farmers (Rich 2020). Chemical controls include synthetic germination stimulant mimics and ethylene to induce suicidal germination, as well as seed coatings with herbicides and chemicals that inhibit strigolactone (SL) biosynthesis (Jamil et al. 2021). However, these chemicals are generally unaffordable to smallholder farmers, their application can be challenging with unpredictable rainfall, and they pose environmental risks (Rich 2020, Jamil et al. 2021). Some sorghum and millet genotypes show partial resistance to *Striga*, but truly resistant lines do not yet exist. In sorghum, a mutation in *LGS1* (*low germination stimulant 1*) (Sibhatu 2016, Gobena et al. 2017) alters the root exudate profile, shifting from the production of a highly active to a weaker *Striga* germination stimulant, thereby reducing *Striga* germination (Gobena et al. 2017). Given that no single strategy is fully effective or always available for smallholder farmers, integrating multiple control methods remains the most promising approach for sustainable control of parasitic weeds.

Beyond conventional methods, microbial control agents have increasingly gained attention as a sustainable and complementary approach for parasitic plant management. The use of microorganisms for controlling parasitic plants has traditionally focused on fungal pathogens that specifically target these parasites. For example, *Fusarium oxysporum* f. sp. *strigae* (Fos) has been shown to specifically infect *Striga* spp. (Elzein et al. 2008). More recently, however, several authors have also proposed the use of beneficial microorganisms as biocontrol agents (Ahonsi et al. 2002, Bouraoui et al. 2012, Mounde et al. 2015, 2020). There is increasing evidence that microbiota, particularly root and rhizosphere communities, play a crucial role in plant growth and health (Mendes et al. 2013). Collectively, these beneficial microbiota can be called plant growth-promoting rhizobacteria (PGPR) and fungi (PGPF) (Yu et al. 2019). These PGPR and PGPF produce phytohormones, assist with nutrient uptake, help plants tolerate stressful environments, and enhance plant defenses against pathogens (Du et al. 2020). Consequently, microbiota—both those that promote plant growth (PGPR and PGPF) and those that do not—can be harnessed to suppress parasitic plant infections (Evidente et al. 2011, Samejima and Sugimoto 2018, Masteling et al. 2019, Mohammadi 2019, Bekele 2020, Qasem 2021, Olowe et al. 2023, Osman et al. 2023). In this review, we discuss the biology of parasitic plants, with a particular focus on how they use chemical host cues to coordinate their lifecycle, and how pathogenic microorganisms, PGPR, and PGPF can interfere with these processes to control parasitic weeds.

## Biology of Parasitic Plants

**Figure 1:**
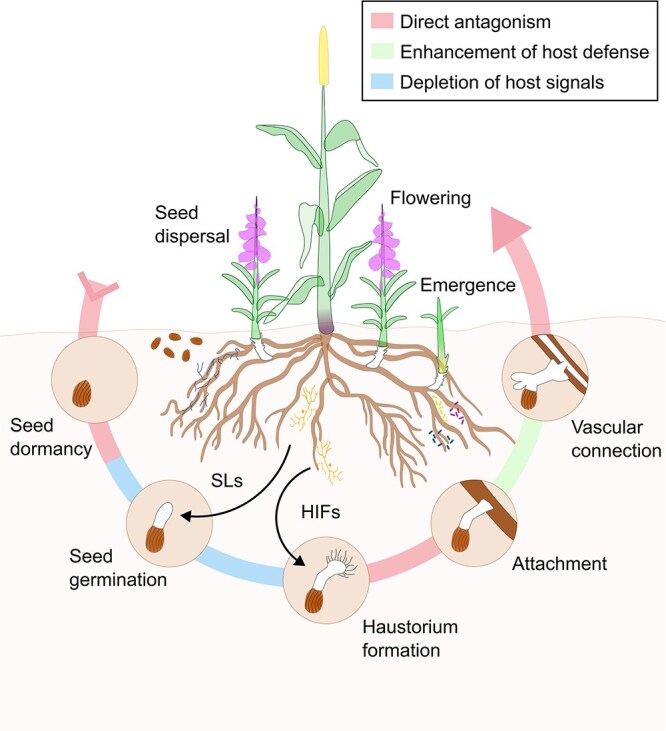
Lifecycle of parasitic plants and how root microbiota suppress them at key stages. The lifecycle of parasitic plants involves several phases. After seed dispersal, seeds remain dormant in the soil until germination is triggered by signaling molecules from host roots. Following germination, haustorium formation occurs in response to additional host-derived signaling molecules, allowing the parasite to attach to the host root. A vascular connection is then established to siphon nutrients and water from the host. Finally, the parasite emerges aboveground, flowers, and produces seeds to continue the cycle. The root microbiota suppresses parasitic plants through three mechanisms. Direct antagonism (red): pathogenic microbes, PGPR, and PGPF inhibit parasitic seeds and belowground establishment through enzymatic degradation and/or production of toxic metabolites. Enhancement of host plant defense (green): PGPR induce physical and biochemical defense responses in the host plant, limiting parasite attachment and vascular connection. Depletion of host-derived signals (blue): PGPR and PGPF degrade and/or reduce exudation of host-derived signaling molecules such as SLs and HIFs, disrupting parasite germination and haustorium formation. Figure created using Inkscape version 1.4.2 (www.inkscape.org).

The lifecycle of parasitic plants (Fig. 1) begins once their seed dormancy is broken as a result of suitable environmental conditions (López-Granados and García-Torres 1996). The host–parasitic plant interaction starts when these seeds germinate upon exposure to allelochemicals exuded by the host plant, termed germination stimulants (Cook et al. 1966, Matusova et al. 2005). After germination, the radicle grows toward the host root, directed by a gradient of these germination stimulants (Ogawa et al. 2022). Upon exposure to another group of signaling molecules exuded by the host plant, termed haustorium inducing factors (HIFs), radicles start forming haustorial hairs (Yoshida et al. 2016, Goyet et al. 2019). If the radicles are close enough, their haustorial hairs enable attachment to and penetration of the host roots (Cui et al. 2016, Auriac et al. 2024). After establishing the connection to the host plant’s vascular system, they can withdraw water and nutrients necessary for survival and growth (Dörr 1997). The parasitic plant then develops further and eventually emerges aboveground, where it flowers and disperses seeds that accumulate in the soil, allowing the lifecycle to begin again (Bouwmeester et al. 2021, Mutuku et al. 2021).

**Figure 2:**
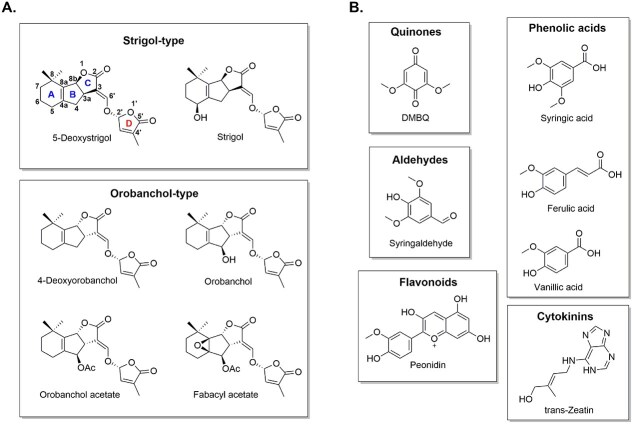
Structures of signaling molecules derived from host plants that regulate key stages in the parasitic plants’ lifecycle. (A) Structures of SLs (Bouwmeester et al. 2021). (B) Structures of HIFs (Goyet et al. 2019, Mutuku et al. 2021).

The germination of obligate parasitic plants depends completely on host-derived signaling molecules. The majority of germination stimulants belong to the group of compounds called strigolactones (SLs, Fig. 2A). SLs function endogenously as hormones that regulate plant development and architecture. The original evolutionary role of SLs, when exuded from roots, is to serve as allelochemicals that promote symbiosis with arbuscular mycorrhizal fungi, a PGPF, in response to environmental changes (Akiyama et al. 2005). Apart from stimulating parasitic seed germination outside the roots, exogenous SLs also act as signals to detect the presence of neighboring plants (Al-Babili and Bouwmeester 2015, Kee et al. 2023, Daignan-Fornier et al. 2024). All natural SLs are derived from carotenoids and contain the C2’(*R*) *α*,*β*-unsaturated furanone moiety (D ring) as shown in Fig. 2A. They are classified into two types based on their structures: the canonical type contains the tricyclic lactone moiety (ABC rings, Fig. 2A) while the non-canonical types do not (Bouwmeester et al. 2021, Soto-Cruz et al. 2021). Canonical SLs can be further classified into strigol- and orobanchol-type SLs (Fig. 2A), where the B-C ring junction of the former is *β*-oriented and the latter is *α*-oriented (Yoneyama et al. 2009, Shimels et al. 2025).

The specificity of parasitic plants toward their host is, partially, determined by the kinds of SLs that the host exudes (Bouwmeester et al. 2021). For instance, *Orobanche foetida* germinates in the presence of the orobanchol-type fabacyl acetate (Fig. 2A), which is found in the root exudate of its native host, faba bean, but not strigol (Fig. 2A; Xie et al. 2009, Fernández-Aparicio et al. 2011). In contrast, *Orobanche cumana* and *Orobanche densiflora* germinate in the presence of strigol, but not fabacyl acetate (Fernández-Aparicio et al. 2011). Furthermore, *Striga hermonthica* has a tendency to germinate more readily in the presence of strigol-type SLs, present in the root exudate of sorghum, whereas *Striga gesnerioides* has a preference for orobanchol-type SLs, produced by its host cowpea (Yoneyama et al. 2009, Shimels et al. 2025). As a result, sorghum cultivars producing orobanchol instead of 5-deoxystrigol (Fig. 2A) showed *S. hermonthica* resistance (Gobena et al. 2017). *S. hermonthica* collected from a sorghum host also germinated less well when exposed to pearl millet root exudate containing orobanchol-type SL (Bouwmeester et al. 2021).

Another class of signaling molecules essential for successful parasitism are the HIFs (Fig. 2B), which are also derived from the host plant. Molecules that induce haustoria include the potent 2,6-dimethoxybenzoquinone (DMBQ, Fig. 2B), isolated from sorghum root extracts, and several other structurally similar phenolic acids (syringic acid, vanillic acid, and ferulic acid), aldehydes (syringaldehyde), and flavonoids (peonidin) as shown in Fig. 2B (Bian et al. 2023). The methoxy moiety adjacent to the hydroxy group on the benzene ring of these compounds facilitates a one-electron redox cycling, which is required for the induction of haustoria (Wang et al. 2019). The methoxyphenol/methoxyquinones likely arise from the degradation of lignin in the host’s cell walls and/or are by-products of lignin polymerization (Mutuku et al. 2021). Compounds structurally unrelated to DMBQ, such as the phytohormone cytokinins (zeatin, Fig. 2B; Aoki et al. 2022), have also been reported to induce haustoria in obligate parasitic plants (Goyet et al. 2019).

### Microbe-mediated mechanisms for parasitic plant control

The mechanisms by which microorganisms can interfere with the host–parasitic plant interaction involve direct antagonism of parasitic plants, the enhancement of host plant defense and the depletion of host-derived signaling molecules (Fig. 1), which will be discuss in detail in the following sections.

#### Direct antagonism of parasitic plants

Early efforts focused on isolating pathogenic fungi from diseased plants to find natural antagonists to control parasitic weeds. This approach led to the isolation of *F. oxysporum* from diseased *O. cumana* in sunflower fields (Bedi and Donchev 1991). Soil inoculation with this isolate resulted in significant suppression of *O. cumana* (Bedi and Donchev 1991, Zwanenburg et al. 1994). Subsequent studies showed that fungal activity reduced seed germination (Thomas et al. 1998), primarily through direct penetration and enzymatic degradation of dormant seeds (Thomas et al. 1999). Fungal germ tubes penetrate the seed coat and colonize internal tissues, where hydrolytic enzymes degrade cell walls and metabolize stored lipids and proteins. While *F. oxysporum* has been the most extensively studied, it is not the only species capable of degrading dormant seeds. *Aspergillus alliaceus*, for example, effectively penetrated and colonized *Orobanche cernua* seeds, resulting in complete degradation of seed contents (Aybeke et al. 2014). By targeting and degrading dormant seeds, pathogenic fungi present a promising strategy for depleting parasitic weed seed banks in infested soils.

Building on this approach, researchers have also identified pathogenic fungi that colonize and degrade later developmental stages of parasitic plants. A well-studied example is the Fos isolate Foxy 2, isolated from diseased *S. hermonthica* in Ghana (Abbasher et al. 1995). This isolate was shown to be highly pathogenic (Kroschel et al. 1996), significantly reducing the emergence of *S. hermonthica* and *Striga asiatica* without affecting other *Striga* species or non-target plants (Elzein and Kroschel 2006). Anatomical studies revealed that, although *Striga* seedlings had formed continuous xylem connections with host sorghum roots, Foxy 2 inhibited further development (Ndambi et al. 2011). The fungus invaded and digested all *Striga* seedling tissues belowground, including haustorial structures, vessels, and cortical parenchyma. In emerged *Striga* plants, fungal hyphae colonized the xylem vessels, impairing water transport, leading to wilting and death of aboveground tissues. Additional Fos isolates with similar effects have been identified, including the Malian isolate M12-4A (Ciotola et al. 1995), Nigerian isolate PSM-197 (Marley et al. 1999), and Kenyan isolate FK3 (Kangethe et al. 2016). Similarly, Boari and Vurro (2004) demonstrated that *F. oxysporum* isolated from diseased *Orobanche ramosa* in Italy invaded seedlings, causing tubercle necrosis without damaging tomato host roots, thus reducing parasitic weed emergence (Boari and Vurro 2004). The ability of these fungi to target multiple life stages of parasitic plants, combined with host specificity, reinforces their potential as effective biocontrol agents.

**Figure 3:**
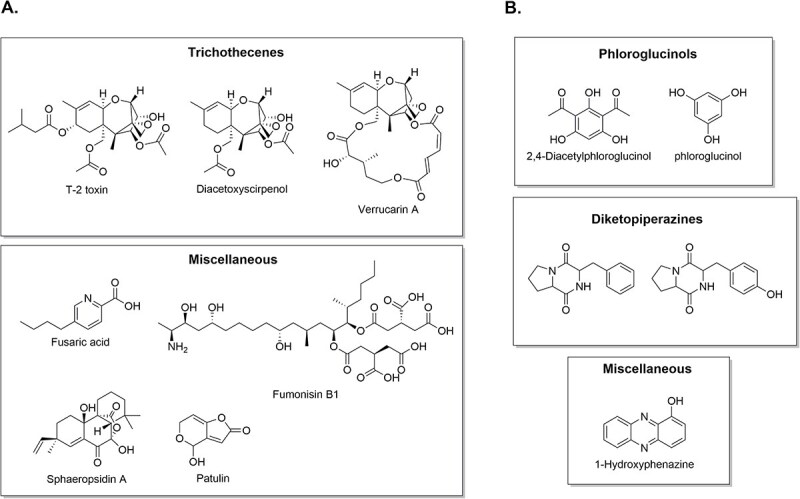
Structures of microbial secondary metabolites that inhibit parasitic plants. (A) Fungal metabolites (Zonno and Vurro 2002, Andolfi et al. 2005, Cimmino et al. 2014, Chen and Jiang 2017, Anteyi et al. 2022). (B) Bacterial metabolites (He et al. 2022, Lurthy et al. 2023, Lurthy et al. 2025).

In addition to enzymatic degradation, fungi also suppress parasitic weeds through the production of toxic secondary metabolites. Several phytotoxic compounds produced by *Fusarium* species have been reported to completely inhibit seed germination of *S. hermonthica in vitro*, including T-2 toxin and deoxynivalenol, fusaric acid and its derivatives, fumonisin B1, and various trichothecenes (Fig. 3A; Vischetti and Esposito 1999, Zonno and Vurro 1999, Idris et al. 2003, Kroschel and Elzein 2004). A recent study identified diacetoxyscirpenol (Fig. 3A) as particularly effective in preventing *S. hermonthica* incidence without adversely affecting sorghum biomass or soil microbial abundance (Anteyi et al. 2022). Interestingly, Fos strains (Foxy 2 and FK3) do not produce this compound, whereas the non-pathogenic *Fusarium venenatum* O86 did, suggesting it could serve as a complementary biocontrol agent targeting seed germination, while Fos targeted germinated or attached seedlings. In addition to *Striga,* germination of *O. ramosa* can also be completely inhibited by toxic metabolites such as T-2 toxin, HT-2 toxin, nivalenol, neosolaniol, and diacetoxyscirpenol (Zonno and Vurro 2002). Other fungal genera including *Myrothecium*, *Diplodia*, and *Seiridium*, produce macrocyclic trichothecenes (e.g. verrucarin A) and tetracyclic pimarane diterpenes (e.g. sphaeropsidin), as shown in Fig. 3A, with similar toxic effects on *Orobanche* and *Phelipanche* spp. (Andolfi et al. 2005, El-Kassas et al. 2005, Cimmino et al. 2014). Beyond pathogenic fungi, well-known PGPF also show promise as biocontrol agents by producing toxic metabolites while supporting host plant growth. For example, *Trichoderma* spp. suppress *Orobanche crenata* and enhance faba bean development (El-Dabaa and Abd-El-Khair 2020). Culture filtrates of *Trichoderma harzianum* inhibit *S. hermonthica* germination, likely due to secondary metabolite production (Hassan et al. 2019, Azarig et al. 2020). Similarly, *Penicillium griseofulvum* produces patulin (Fig. 3A), a mycotoxin that suppresses the germination of *O. cumana* and *Phelipanche aegyptiaca* (Chen et al. 2017). Altogether, these findings highlight the potential of fungi that produce toxic metabolites and degradative enzymes as effective biocontrol agents for selective suppression of parasitic weed germination and belowground establishment.

Besides secondary metabolites, certain primary metabolites have been shown to suppress parasitic plants. High concentrations of amino acids leucine and tyrosine are toxic to *S. hermonthica* but not maize, as they interfere with amino acid biosynthesis in *Striga* through feedback inhibition (Sands and Pilgeram 2009, Nzioki et al. 2016). In addition, methionine can enhance the virulence of *F. oxysporum* by being metabolized into ethylene, which stimulates *Striga* seed germination (Logan and Stewart 1991) and leads to the formation of seedlings that are more susceptible to infection (Nzioki et al. 2016). Hence, harnessing *F. oxysporum* variants capable of overproducing all three of these amino acids significantly enhance the pathogenicity of the fungi against *Striga* (Nzioki et al. 2016). Similarly, amino acids proline, arginine, histidine (Vurro et al. 2009), as well as methionine (Fernández-Aparicio et al. 2017), have been reported to inhibit germination and radicle elongation of *Phelipanche ramosa* and *O. crenata*. Collectively, these findings demonstrate the potential of fungi producing specific primary metabolites to function autonomously as biocontrol agents or to synergistically enhance the efficacy of other fungal agents within a mixture.

In addition to fungi, certain bacteria, primarily PGPR, also suppress parasitic weeds through enzymatic degradation, the production of toxic metabolites or phytohormones, and the release of inhibitory volatiles. Bacterial isolates from *Striga*-suppressive soils in Kenya, mainly belonging to the genus *Bacillus*, degrade *S. hermonthica* seeds through the secretion of various hydrolytic enzymes (Neondo et al. 2017). A strain of *Bacillus velezensis*, a well-known PGPR, produces diketopiperazine-type metabolites (Fig. 3B) that completely inhibit the germination of *Orobanche aegyptiaca* seeds while simultaneously enhancing tomato growth (He et al. 2022). Similarly, several *Pseudomonas* strains produce germination-inhibiting compounds such as 1-hydroxyphenazine and phloroglucinol (Fig. 3B), which suppress *Orobanche* species (Lurthy et al. 2025). Specifically, *Pseudomonas ogarae* synthesizes 2,4-diacetylphloroglucinol (Fig. 3B), which irreversibly blocks the germination of *P. ramosa* (Lurthy et al. 2023). In addition, volatile sulfur-containing organic compounds have been identified as effective inhibitors of parasitic weeds. *Burkholderia* species produce dimethyl disulfide, which has demonstrated strong control of *P. aegyptiaca* (Joel et al. 2013) and was recently approved for use against *Orobanche* spp. in Turkey (Kûkürt et al. 2024). *Bacillus* sp., *Burkholderia pyrrocinia*, and *Paraburkholderia* spp., as well as co-inoculation cultures of *Burkholderia vietnamiensis* with *T. harzianum*, have also been reported to produce dimethyl disulfide (Meldau et al. 2013, Carrión et al. 2018, Li et al. 2024). Alternatively, bacteria from the genera *Klebsiella*, *Pseudomonas*, *Bacillus*, and *Enterobacter* have shown suppressive activity against *Striga* by producing auxins that interfere with haustorium formation (Keyes et al. 2000, Mohammadi 2019, Tulu et al. 2024). Other PGPR that produce unidentified bioactive compounds with inhibitory effects on parasitic plants have also been reported. For instance, lipophilic extracts from *Azospirillum brasilense* culture medium suppressed *S. hermonthica* germination (Miche et al. 2000), while cell-free culture filtrates of *Streptomyces enissocaesilis* and *Streptomyces pactum* inhibited seed germination of *O. cumana* and *P. aegyptiaca*, respectively (Chen et al. 2016, 2020). Sometimes the exact mechanism of inhibition remained unclear, as observed in several PGPR strains from the genera *Bacillus*, *Pseudomonas*, *Streptomyces*, and *Azospirillum* that inhibited seed germination and radicle elongation of parasitic weeds while simultaneously promoting host plant growth (Bouillant et al. 1997, Zermane et al. 2007, Hassan et al. 2011, Mounde et al. 2015, El-Dabaa and Abd-El-Khair 2020, Iasur Kruh et al. 2020, Osman et al. 2024). Taken together, these insights reveal the potential of PGPR and other bacteria as sustainable bioherbicides.

#### Enhancement of host plant defense

Another microbe-mediated mechanism to control parasitic plants is through the enhancement of host defense. This has been illustrated in host plants inoculated with *Rhizobium* strains, which exhibited upregulation of defense-related enzymes such as peroxidase, phenylalanine ammonia lyase (Y. Mabrouk et al. 2007b, 2016), and polyphenol oxidase (Mabrouk et al. 2010). Elevated expression of these enzymes altered cell wall composition by enhancing lignification, suberization, and xylem occlusion at the infection site, thereby preventing haustorial penetration or restricting nutrient translocation to the parasite if vascular connections are established (Passardi et al. 2005, Y. Mabrouk et al. 2007b, 2016). Similarly, *Bacillus amyloliquefaciens*, *Bacillus pumilus*, and *Pseudomonas fluorescens* have been shown to increase peroxidase activity in tomato and pepper (Ramamoorthy et al. 2002, Jetiyanon 2007). The enzymatic activity of polyphenol oxidase also increased following application of *S. enissocaesilis* to sunflower roots, suggesting its role in enhancing host defense (Chen et al. 2016). In sorghum, *Arthrobacter* was shown to promote endodermal suberization, forming a physical barrier that impedes *Striga* intrusion (Kawa et al. 2024). Inoculation of *Rhizobium leguminosarum* in faba bean has been shown to significantly decrease the germination and emergence of *O. foetida* (Bouraoui et al. 2012), possibly by inducing the host’s exudation of phenolic compounds toxic to *Orobanche* seeds, as demonstrated in pea inoculated with *R. leguminosarum* (Y. Mabrouk et al. 2007a). Similar protective effects have been observed in chickpeas against both *O. foetida* (Hemissi et al. 2013) and *O. crenata* (Mabrouk and Belhadj 2014), likely due to toxic metabolites secreted by host roots. Together, these examples underscore how microbial agents can reprogram the host plant to enhance its self-protective mechanisms against parasitic plants.

#### Depletion of host-derived signals

Microorganisms can also disrupt host–parasitic plant interactions by downregulating the production of host-derived signaling molecules. Host plants secrete SLs (Fig. 2A) to initiate the symbiosis with arbuscular mycorrhizal fungi (AMF); interestingly, co-cultivation with AMF results in reduced SL production through an autoregulation mechanism to avoid over-colonization (Mitra et al. 2021). Furthermore, once AMF successfully improves nitrogen and phosphorus availability, SL exudation typically declines (Yoneyama and Brewer 2021). Supporting this concept, root exudates from sorghum colonized by AMF (*Glomus clarum* and *Gigaspora margarita*), induced less *S. hermonthica* germination compared to exudates from non-mycorrhizal sorghum (Lendzemo et al. 2007). Likewise, *Striga* incidence and biomass were reduced upon AMF inoculation in maize (Othira et al. 2012). A similar effect was observed with pea root exudate colonized by AMF (*Glomus mosseae* and *Glomus intraradices*), which reduced germination of *Orobanche* and *Phelipanche* (Fernández-Aparicio et al. 2010). Root exudates of tomato colonized by AMF showed lower levels of orobanchol-type SLs compared to exudates from non-colonized roots (López-Ráez et al. 2011). Together, these findings demonstrate that AMF can both enhance nutrient availability and modulate host signaling, thereby suppressing parasitic weed germination.

Beyond mycorrhizal associations, SLs have been implicated in the *Rhizobium*-legume symbiosis, recruiting rhizobia and promoting formation of nitrogen-fixing nodules on the roots of legume plants (Soto et al. 2010, Clark and Bennett 2024). In alfalfa nodulated with *Sinorhizobium meliloti*, reduced biosynthesis of orobanchol and orobanchyl acetate (Fig. 2A) was observed, resulting in decreased *P. ramosa* seed germination (Peláez-Vico et al. 2016). Similarly, (Y. Mabrouk et al. 2007a) suggested that inoculation of *R. leguminosarum* strains onto pea roots conferred protection against *O. crenata* by reducing the exudation of germination stimulants from host roots (Y. Mabrouk et al. 2007b). These findings suggest that rhizobia can be harnessed not only to enhance nitrogen availability but also to reduce the exudation of host signals and suppress parasitic weed germination.

In addition to symbiotic microbes, other soil-dwelling bacteria and fungi have shown the capability to degrade SLs and disrupt parasitic seed germination. In an experiment conducted by Ali et al. (2013), the addition of a *Pseudomonas* sp. suspension to sorghum root exudates significantly reduced the exudates’ ability to induce *Striga* seed germination. This effect has been attributed to the production of enzymes, such as esterases or lactonases, by the *Pseudomonas* sp., which hydrolyzed the D ring of SLs, leading to their inactivation and subsequent disruption of host–parasitic plant signaling (Ali et al. 2013). The presence of hydrolases similar to α/β-fold hydrolases, responsible for the hydrolysis of natural SLs in plants (De Saint Germain et al. 2016), have been identified in *Bacillus subtilis* (Quinodoz et al. 2023, Melville et al. 2024). Fungal strains, including *T. harzianum* and *F. oxysporum*, have also shown the ability to degrade SLs such as 5-deoxystrigol and 4-deoxyorobanchol (Boari et al. 2016). These findings highlight the potential of microorganisms to degrade germination stimulants, offering a promising biological approach to control parasitic weeds by preventing seed germination.

Given that various HIFs have already been identified (Goyet et al. 2019), exploiting microbes capable of degrading these signals offers an alternative way to disrupt the host–parasitic plant interaction. By preventing haustorium formation, germinated seeds cannot attach to the host and will die within several days, a process called suicidal germination, which will result in a reduction of the seed bank. For example, *Pseudomonas* species have been shown to decrease the content of phenolics in sorghum root exudate (Ali et al. 2013). In a recent study, a *Pseudomonas* strain isolated from Dutch soil was able to degrade syringic acid, resulting in reduced *Striga* haustorium formation (Kawa et al. 2024). Similarly, *Sphingobacterium* species have been shown to efficiently degrade phenolic compounds such as vanillic acid (Fig. 2B), *p*-coumaric, and syringic acid (Wang et al. 2018, Hagaggi and Abdul-Raouf 2022), making them interesting candidates for application in parasitic plant systems. Thus, disrupting HIF signaling through microbial degradation represents a promising strategy for managing parasitic weeds.

## Field Trials and Practical Applications

Several field trials implementing fungi and bacteria as bioherbicides have been documented. Fos isolates PSM197 and Foxy 2, formulated as pesta granules, have been shown to effectively reduce *Striga* biomass in African maize fields (Venne et al. 2009). The Toothpick Project initially coated toothpicks with a primary inoculum of three Fos variants capable of overproducing the amino acids leucine, tyrosine, and methionine, to facilitate more efficient subsequent propagation. This method successfully reduced *Striga* emergence by 80%–92% and increased maize yields in Kenya by 42%–56% (Nzioki et al. 2016). More recently, the project has commercialized their fungal herbicidal agent in the form of seed coatings, which improved shelf life and provided farmers with greater planting flexibility (Baker et al. 2024). In a field study on maize and sorghum, application of AMF propagules significantly reduced *S. hermonthica* infestation (Lendzemo et al. 2005). Fermentation broth of *B. velezensis* applied to tomato fields was capable of controlling the growth of *O. aegyptiaca* (He et al. 2022). Application of *S. pactum* inoculum in a field experiment reduced *O. aegyptiaca* emergence while simultaneously increasing tomato yield (Chen et al. 2020). In addition, a reduction in *O. foetida* emergence following inoculation with a *R. leguminosarum* strain has been reported in faba bean fields of Tunisa (Bouraoui et al. 2016). These field trials demonstrate that microbial inoculants can effectively mitigate the impact of parasitic plants under practical agricultural conditions.

The potential of microbes has led to the establishment and funding of the PROMISE Project (Promoting Root Microbes for Integrated Striga Eradication, https://promise.nioo.knaw.nl/en). This initiative aims to harness the functional capabilities of soil- and plant-associated microbes (both bacteria and fungi) to reduce yield losses caused by *Striga*. The project is structured around four main research areas. First, it investigates the effects of different organic substrates in stimulating the production of Strigacidal volatile compounds by specific soil microorganisms, which can inhibit *Striga* germination. Second, it aims to develop and apply fungal inoculants capable of reducing the *Striga* seedbank in the soil. Third, the project focuses on formulating bacterial inoculants that diminish the seedbank either by triggering suicidal germination or by promoting decay of *Striga* seeds. In addition, microbial strains capable of degrading germination stimulants and HIFs are being identified. Finally, PROMISE explores the development of microbial inoculants that induce the formation of physical barriers within the host plant root, thereby limiting *Striga* infection and improving plant resistance. Through these integrated approaches, the PROMISE project aims to deliver sustainable, biologically driven solutions for *Striga* control by combining multiple strategies, reducing the seed bank and disrupting chemical signaling between host and parasite, to improve crop yields.

## Conclusion and Future Perspective

Parasitic plants of the Orobanchaceae family, particularly *Striga*, *Orobanche*, and *Phelipanche* spp., represent a major threat to agriculture, causing severe yield losses across multiple continents. Conventional control measures have shown limited success due to economic, environmental, and practical constraints, especially for smallholder farmers. Recent advances reveal that root-associated microbiota, including both pathogenic and beneficial microorganisms, offer promising alternative strategies for the sustainable management of these parasitic weeds.

Microbial biocontrol agents can suppress parasitic plants through multiple mechanisms: direct antagonism via production of toxic metabolites or enzymatic degradation of parasitic plant seeds and seedlings; enhancement of the host plant defense, including induction of physical and biochemical changes; and disruption of the chemical signaling that coordinates parasitism, such as degrading or reducing exudation of SLs and HIFs. The ability of beneficial microbes to simultaneously promote host plant growth and suppress parasitic weeds underscores their dual utility in parasitic plant management.

Despite promising results in controlled environments, widespread field adoption of microbial biocontrol agents faces challenges such as inconsistent efficacy across different soil types and environmental conditions, competition with native microbiota, and formulation or shelf-life limitations, as encountered in the toothpick project. Overcoming these obstacles requires a deeper understanding of the mechanisms underlying microbe–parasite–host interactions, along with optimizing microbial formulations and evaluating their long-term efficacy across diverse field conditions. Multidisciplinary collaboration across microbiology, plant physiology, chemistry, and agronomy will be essential to develop robust, cost-effective, and environmentally sustainable solutions. Ultimately, harnessing the power of root microbiota holds great promise to sustainably control parasitic weeds, improve crop productivity, and support global food security.

## Data Availability

No new datasets were generated or analyzed in this study.
